# EffFeu Project: Towards Mission-Guided Application of Drones in Safety and Security Environments

**DOI:** 10.3390/s19040973

**Published:** 2019-02-25

**Authors:** Christopher-Eyk Hrabia, Axel Hessler, Yuan Xu, Jacob Seibert, Jan Brehmer, Sahin Albayrak

**Affiliations:** DAI-Labor, Technische Universität Berlin, Ernst-Reuter-Platz 7, 10587 Berlin, Germany; axel.hessler@dai-labor.de (A.H.); yuan.xu@dai-labor.de (Y.X.); j.seibert@campus.tu-berlin.de (J.S.); jan.brehmer@dai-labor.de (J.B.); sahin.albayrak@dai-labor.de (S.A.)

**Keywords:** decisional autonomy, decision-making, planning, object recognition, deep learning, GNSS-denied localisation

## Abstract

The number of unmanned aerial system (UAS) applications for supporting rescue forces is growing in recent years. Nevertheless, the analysis of sensed information and control of unmanned aerial vehicle (UAV) creates an enormous psychological and emotional load for the involved humans especially in critical and hectic situations. The introduced research project EffFeu (Efficient Operation of Unmanned Aerial Vehicle for Industrial Firefighters) especially focuses on a holistic integration of UAS in the daily work of industrial firefighters. This is done by enabling autonomous mission-guided control on top of the presented overall system architecture, goal-oriented high-level task control, comprehensive localisation process combining several approaches to enable the transition from and to GNSS-supported and GNSS-denied environments, as well as a deep-learning based object recognition of relevant entities. This work describes the concepts, current stage, and first evaluation results of the research project.

## 1. Introduction

The number of unmanned aerial system (UAS) applications for supporting common firefighters and industrial firefighters is growing in recent years [1,2]. In particular exploration, surveillance, monitoring and documentation of accidents, dangerous places and critical infrastructure are evaluated. Up to this point, most gathered information, for instance, video footage, is analysed manually by trained professionals without any additional support. Moreover, aerial systems do always require a trained pilot, an extra person who is using either semi-automated control based on GNSS (global navigation satellite system) or full manual control in GNSS-denied environments (e.g., indoors or close to environmental structures such as buildings or trees). Such analysis and control creates an enormous psychological and emotional load especially in critical and hectic situations, apart from the fact that needing two extra persons to operate the drone is unacceptable.

Other research projects have already investigated the application of UAS in related areas of civilian safety. The project NExt UAV [3] tested single and multiple UAS in disaster rescue scenarios, and the particular focus lies on navigation and communication of the drones. Other projects in the domain of disaster rescue are ICARUS [4] and SENEKA [5], which aim for terrain exploration and the application of sensor networks. AirShield [6] concentrates on the protection of critical infrastructure through danger prognosis and visualisation of situation information. Here, the information gathering is based on a sensor network with multiple drones that are flying on a priori defined trajectories. Surveying radioactivity pollution with a drone swarm from a greater distance is the aim of ANCHORS [7]. The interaction between the user and the system is researched in ORCHID [8]. In this project, artificial intelligence is applied to manage and control both humans and robots based on gathered information in a disaster scenario.

The related work is concentrating on research about UAS communication, application of sensor networks, and information sensing. A holistic integration into existing IT infrastructure, such as in safety and security management tools, and autonomous application from a mission-oriented perspective is not considered yet. Furthermore, the perceived information is not further processed and annotated to simplify the analysis for the human operator. Moreover, the integrated application of drones in GNSS-denied environments is also missing attention.

In order to address the open points, our project EffFeu enables a mission-guided application of drones that is tightly integrated into a safety and security management system, while reducing the load of manual control and analysis through automated object recognition that is augmented to the video footage by our partners. Moreover, EffFeu targets the challenge of a robust localisation in the transition between environments with reliable GNSS-based localisation and localisation in a priori known but GNSS-denied environments. All parts of the project EffFeu are aiming for a holistic integration of UAS in the daily work of industrial firefighters.

This paper is a revised and extended version of [9]. This extension provides especially additional information about the general architecture, used software components as well as new experiments, insights and details about the object recognition, localisation in the transition between indoors and outdoors as well as mission-guided control components. For the object recognition, we conducted in particular experiments to empirically show the effectiveness of different popular design choices to select the most suitable one for our scenario. The experiments about our localisation process determine the most suitable map alignment approach for our setup as well as if partially available GNSS allows us to improve the overall accuracy. Regarding our mission-guided control component, additional details about the architecture for the User Interface (UI) integration are provided, while complementary experiments study how our solution performs in a more complex scenario with different configurations and dynamically adjusted mission boundaries.

The contributions of this paper are manifold. First, the proposed architecture allows, to our best knowledge, for the first time, the comprehensive integration of drones in an industrial Safety and Security Management System. Secondly, we propose an object-recognition approach for drones tailored to the requirements of industrial firefighters. Thirdly, our localisation and navigation process addresses the problem of operation in partially GNSS-denied environments making use of a priori known information. Fourthly, we present the realisation of a mission-guided control of drones as well as its integration in the overall approach on the foundation of a hybrid decision-making and planning system.

In the remainder of the paper, we first introduce the research project with partners and responsibilities in more detail before we present the overall project system architecture. In the following sections, we provide more details about the mentioned contributions discussing concepts, current stage and first results of the project parts that our department realised.

## 2. Project

The goal of the EffFeu project is the comprehensive integration of UAS into the Safety and Security Management as it is used by industrial firefighters, while increasing the robustness and efficiency through the development of intelligent support functionalities. Portability, openness and extendibility of the solution as well as connectivity, acceptance and application of common standards are consequently considered during the development of the project.

The project is organised into three subprojects, one for each of the three consortial partners: Gemtec GmbH (Königs Wusterhausen, Germany) is dealing with the integration of UAS into the management system and the development of the drone hardware, ART+COM AG (Berlin, Germany) investigates interactive user interfaces for UAS, and Distributed Artificial Intelligence (DAI)-Labor of the Technische Universität Berlin (TU Berlin) extends the intelligence of the UAS. All three partners have a large integration work package where they are enforced to integrate and test their components into the overall system and context.

In detail, the Gemtec subproject is focused on integrating sensory output of drones, its sensor payloads and UAS controls into their safety and security management system WotanEx (http://wotanex.de/loesungen/security.html) so that data and information acquired can be seamlessly used by control room personnel and task force for human decision-making.

The ART+COM subproject works on the augmentation of the drone camera streams with additional relevant information in real time. They also develop user interfaces that guarantee the intuitive and goal-oriented interaction with the drone, either by the control room personnel or the task force on-site.

The subproject of the DAI-Labor focuses especially on objects and situation recognition, autonomous navigation in the transition from absolute GNSS localisation to relative localisation and mapping approaches (e.g., simultaneous localisation and mapping (SLAM)), and the intelligent, application- and mission-oriented control of aerial systems.

The following section introduces the system architecture of the project that reflects the complementary responsibilities of the project partners.

## 3. Architecture

**Figure 1:**
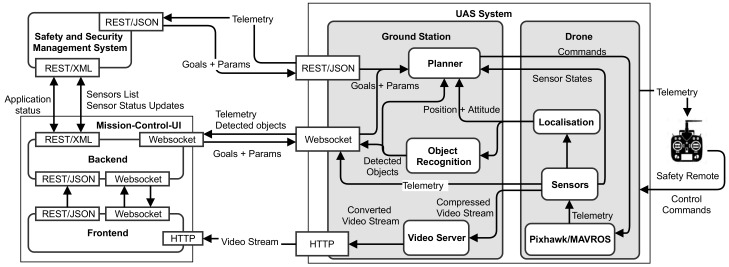
EffFeu system architecture.

The nature of the EffFeu architecture is a holistic structure of a distributed system of distributed systems. It can also be characterised as a cyber-physical system utilising standard communication infrastructures and protocols between mechanical and electronic components, tightly coupled with intelligent algorithms and users. The overall architecture is shown in Figure 1.

The main components of the system are reflecting the responsibilities of the project partners, namely the *Safety and Security Management System*, the The *Mission-Control-UI*, and the *UAS System*.

The *Safety and Security Management System* software allows for integrating, monitoring and controlling various sensors and actors of industrial plants, for instance fire warning devices, video systems, and access control systems. Responsible for the operation of the software are experts that manage occurring alarms and events in a central control room, e.g., on an industrial plant with own fire brigade, like a chemical plant.

The *Mission-Control-UI* is an intuitive and goal oriented user interface that is used by the rescue forces in operation on a ruggedized mobile tablet computer. It allows for selecting and configuring high-level mission goals for the drones and gives access to the automatically gathered information, like recognised objects, as well as the current system state. To manage several parallel clients, the component is split into a *Frontend* and *Backend*.

DAI-Labor is concentrating on the drone and ground station components and the provisioning of interfaces, which can be used by the partners both for monitoring the drone and its sensor outputs as well as for controlling it using high-level mission goals. The *UAS system* consists of the major components: *Planner*, *Object Recognition*, *Localisation*, and *Web Video Server*. Each component is embedded in several Robot Operating System (ROS) processes and can reside and run either on the drone or on the ground station computer.

The *Planner* component is responsible for the task-level decision-making and planning of the system. It is instructed by high-level mission goals that are selected and parametrised in the user interface of the *Safety and Security Management System* component or the *Mission-Control-UI*. The planner interprets the goals and creates the flow of activities that are then translated to low-level drone behaviours and executed. More details are given in Section 4.3.

The *Localisation* component enables a seamless operation of the drones indoor and outdoor based on GNSS information, R-GBD (Red Green Blue-Depth)-based SLAM, visual odometry and prior known environment maps. Section 4.2 provides additional information about our approach.

The *Object Recognition* component is a one-shot multi-object detector for multiple categories and varying resolutions. The pre-trained part may reside on the drone itself, and the online training unit is running on the ground station. According to the firefighter’s priorities, it is capable of detecting persons, vehicles and hazardous goods in real time, also reconstructing the 3D world coordinates of objects in the image. Further details about the object recognition are given in Section 4.1.

The main task of the *ground station* is to provide gateway-like functions such as converting telemetry data and sensor information into needed representations and providing them over different interfaces. The function of the ground station is bilateral, also incoming control messages will be translated and delivered to according components.

The *Safety Remote* is only a backup control instance that allows taking over control in case the system behaves maliciously aside from being currently required for regulatory reasons.

The drone itself is controlled on low-level by a Pixhawk autopilot system with a PX4 software stack that is instructed by the *Planner*. All higher-level components of the UAS, including Planner, Localisation, and Object Recognition are implemented with ROS. The ROS component instances, so-called nodes, can be freely distributed between the ground station computer and the drone itself. The ROS nodes on the drone are running on a comparable powerful small-size x86 companion computer (Intel^®^ NUC7i7BNH) that has a serial connection with MAVLink protocol to the Pixhawk. The automated conversion between MAVLink commands and the ROS communication infrastructure is realised with the MAVROS (http://wiki.ros.org/mavros) package. In order to provide REST/JSON, Websocket, and HTTP video stream interfaces, we make use of the ROS packages ROStful (https://github.com/pyros-dev/rostful), Rosbridge suite (http://wiki.ros.org/rosbridge_suite), and a customised and extended version of the web_video_server (https://gitlab.tubit.tu-berlin.de/breakdowncookie/web_video_server). These packages allow us to automatically generate required interfaces for the partner systems based on an Application Programming Interface (API) we conveniently have defined with standard ROS functionalities.

The novelty of the project architecture is the realisation of a holistic integration of UAS based on ROS with additional components that are required to achieve a seamless and mission-oriented workflow for rescue forces. Here, the additional components from the perspective of the UAS are the Safety and Security Management System and the Mission-Control-UI. To our best knowledge, this is the first time that an industrial Safety and Security Management System is equipped with means for controlling and monitoring drones in operation. Furthermore, the integration with the Mission-Control-UI is different to existing control approaches because it entirely focuses on a mission-oriented and task-based perspective instead of a traditional motion-oriented control.

## 4. UAS System Details and Results

In the following subsections, we present the approaches, first results and insights about the core research topics of the DAI-Labor within the EffFeu project that are focussing on the realisation of an intelligent UAS system.

### 4.1. Object Recognition

Object detection is the problem of finding and classifying a variable number of objects on an image. With the development of deep learning, backed by big training data and advanced computing technology, the ability of immediately recognizing all the objects in a scene seems to be no longer a secret of evolution. One popular approach is Region-based Convolutional Neural Network (R-CNN) framework [10], which is based on a two-stage, proposal-driven mechanism. Through a sequence of advances, this two-stage framework consistently achieves top accuracy on the challenging COCO [11] benchmark. On the other hand, one stage detectors are applied over a regular, dense sampling of object locations, scales, and aspect ratios. Recent work on one-stage detectors, such as *You Only Look Once* (YOLO) [12] and *Single Shot MultiBox Detector* (SSD) [13], demonstrates promising results, yielding faster detectors with lower accuracy. These algorithms are not usually optimal for dealing with sequences or images captured by drones, due to various challenges such as viewpoint changes and scales. In this project, we have developed a modular object recognition based on deep convolutional neural networks to experiment and tune different submodules for images captured by drones. There was no big dataset available for drone-based object recognition applications until recently [14,15]; therefore, we have applied transfer learning, e.g., our model firstly was trained with the big dataset (COCO), and then fine-tuned with the small drone dataset, which is created in house.

#### 4.1.1. Architecture of Object Recognition Module

**Figure 2:**
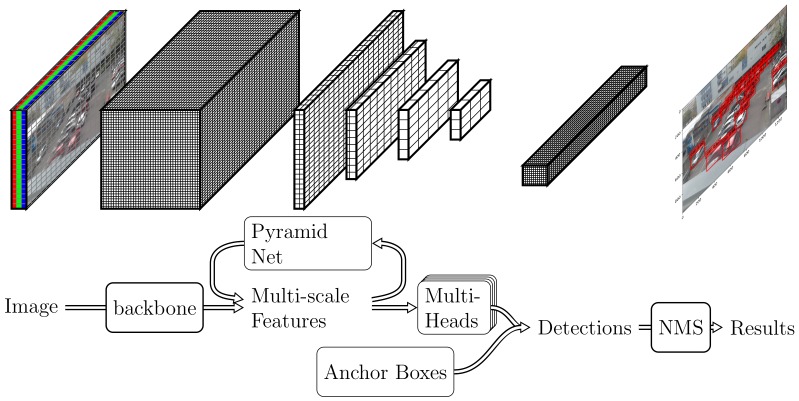
Meta architecture of one stage deep Convolutional Neural Network (CNN) based object recognition. (**Top**): Tensor data representation in different steps. (**Below**): Submodules and processing steps, see text for more details.

Considering computation limits, one stage deep convolutional neural network based method is chosen, our development was started with the static graph framework [16], and switched to PyTorch [17] for dynamic graph support, which allows us to enjoy fast prototyping and GPU computation. The software evolves from monolithic to modular as well, the basic concepts of SSD becomes the meta-architecture, as illustrated in Figure 2. Different submodules are developed and tested following recent deep learning advances, specifically [10,18]:

**backbone net** is responsible for computing a convolutional feature map over an entire input image and is an off-the-self pretrained convolutional network. We have choices between VGG [19], ResNet [20], ResNext [21], and SE-ResNet [22] for achieving the right speed/memory/accuracy balance [23].
**pyramid net** augments a standard convolutional network with a top-down pathway and lateral connections so the network efficiently constructs a rich, multi-scale feature pyramid from a single resolution input image. LateralBlock [24], ThreeWayBlock [25], and TransferConnectionBlock [26] have been implemented.
**anchor boxes** associates every feature map cell to a default bounding boxes of different dimensions and aspect ratios. These are manually chosen for different applications.
**multi-heads net** predicts the probability of object presence at each spatial position for each of the anchors and object classes. Bottle Net Block [20], DeformbleConv [27] and Receptive Field Block [28] have improved over basic convolutional block.
**post processing** merges and applies non-maximum suppression to all detections to yield the final results.

#### 4.1.2. Implementation and Experiments

While our implementation has the flexibility to combine different submodules, e.g., backbone, pyramid, etc., it is not clear which combination achieves the right speed/memory/accuracy balance for our application and platform. Previous work typically only states that they achieve some accuracy with some frame-rate but do not give a full picture of the speed/accuracy trade-off, which depends on many other factors, such as which framework is used, which post-processing is used, etc. Though it is impractical to evaluate every combination of our submodules, we are fortunate that many of them can be excluded by desired speed and accuracy. In this paper, we seek to explore the speed/accuracy trade-off of different submodules in an exhaustive and fair way.

In order to experiment with different configurations, we investigated techniques which train neural network faster. In the end, we use AdamW [29] as an optimizer, it improves Adam’s generalization performance and trains much faster than the stochastic gradient descent (SGD). Mixed-Precision training [30] is also used in new Nvidia GPUs, and it decreases the required amount of memory and shortens the training.

**Figure 3:**
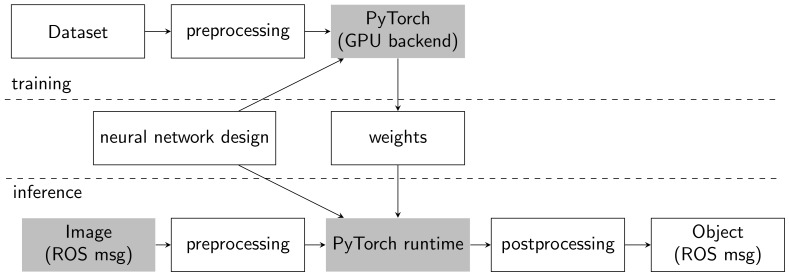
Object detection training and inference pipelines.

The training and inference pipelines of our detection system are shown in more detail in Figure 3.

Inspired by [23], experiments are taken to empirically show the effectiveness of different design choices. We conduct training and evaluation on the Microsoft COCO dataset [11]. This dataset involves 115,000 training images and 5000 validation images as recommended train/eval split in 2017. The predictions of models are evaluated by *Intersection over Union* (IoU), which is essentially a method to quantify the percent overlap between the target bounding box and prediction output. The overall *Mean Average Precision* (mAP) over different IoU thresholds from 0.5 to 0.95 (official COCO metric, or mAP@0.5:0.95) is used for model evaluation, and mAP at single threshold 0.5 (official Visual Object Classes (VOC) metric, or mAP@0.5) as well. The COCO metric emphasizes better bounding boxes than classification accuracy, but it has its weakness in practice: the dataset can have inaccuracies in bounding boxes due to human labelling. On the other hand, classification accuracy is more important than bounding boxes in real applications where false detection can be a disaster and rough bounding boxes can be tolerant. This is especially relevant in the potentially dangerous scenario addressed in the EffFeu project.

All the models are trained with exactly the same setup except the backbone because we want to keep a similar setup as [18] for comparing the performance of our implementation with a known record. The input images are resized to 512×512, varies of data augmentations were used, including horizontal flip, random crop, and photometric distort which randomly adjusts brightness, contrast, saturation and hue.

**Figure 4:**
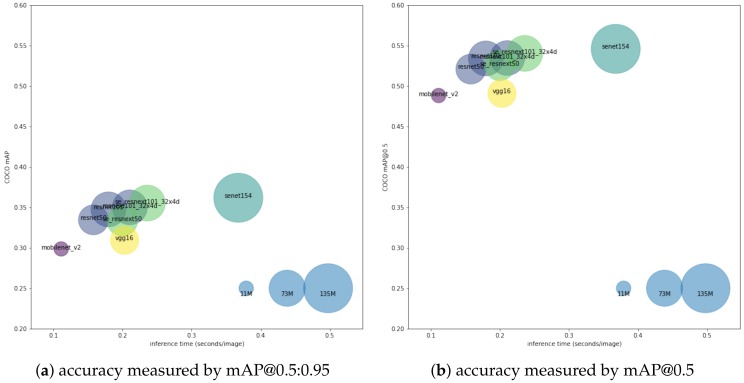
Accuracy vs. memory vs. time, with marker colours indicating backbone net and size indicating number of parameters. The accuracy metrics are different in (**a**) and (**b**): mean average precision over different IoU threshold from 0.5 to 0.95 is used in (**a**), and mean average precision with IoU threshold at 0.5 is used in (**b**).

Figure 4 shows scatterplots visualizing the results of models trained 100 epochs on COCO dataset, colours indicating backbone net, and size indicating number of parameters. The inference benchmark was done with Nvidia Titan Black GPU, Intel i7 4 GHz CPU, and 16 G RAM. Generally, we observe that a bigger backbone network achieves better accuracy, while trading off the speed. Furthermore, detailed results in Table 1 show that: (1) very small network (such as mobilenet_v2) speed is limited by post-processing; (2) bigger networks improve both bounding boxes and classification accuracy as shown in mAP@0.5:0.95 and mAP@0.5; (3) the model with resnet50 as backbone achieves 0.52 mAP@0.5 with 6.3 frames per second, and we identify it is the sweet spot on the accuracy/speed trade-off curve.

#### 4.1.3. Results on Drone Dataset

**Figure 5:**
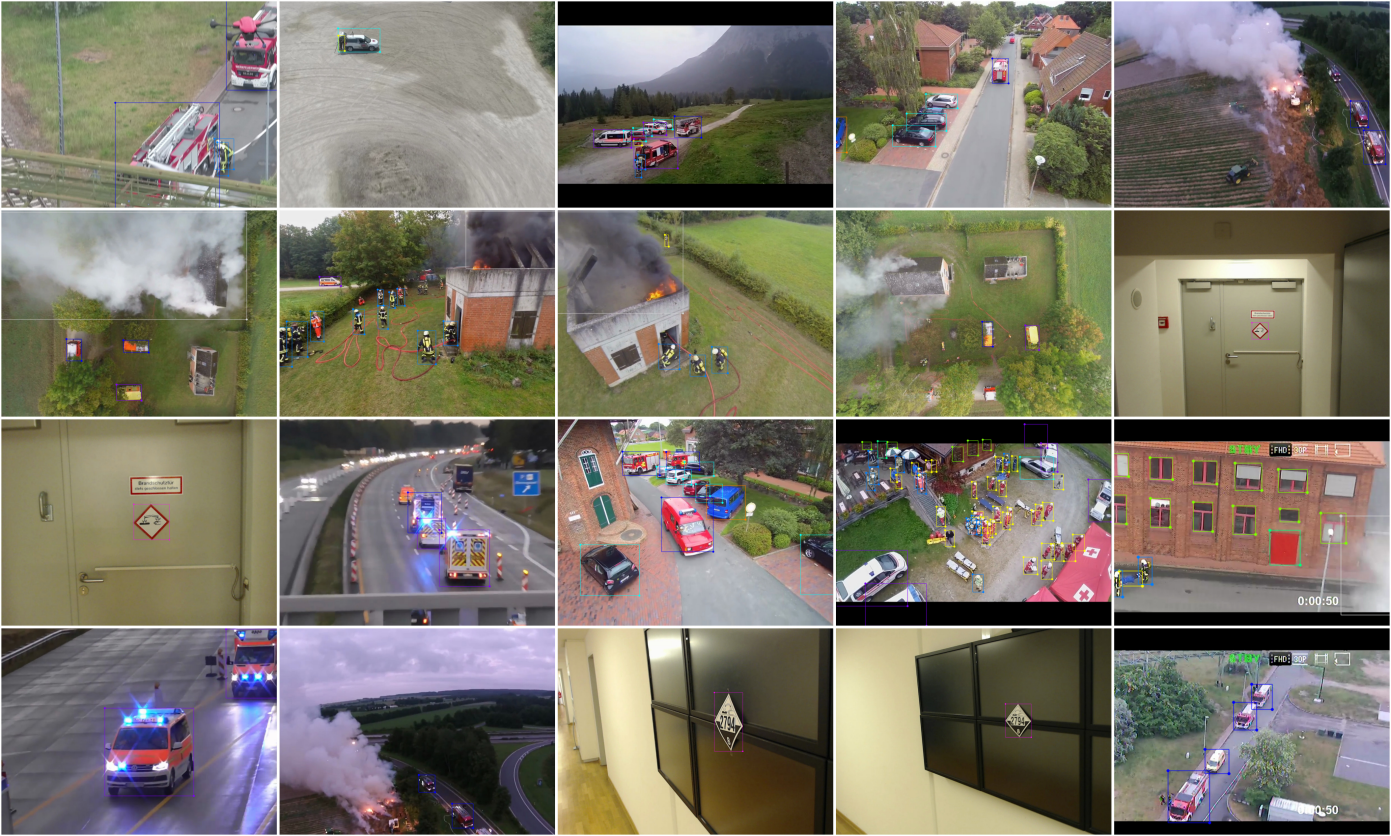
Example images and labels of EffFeu project-specific dataset.

For the EffFeu project, the three most important classes of objects are vehicle, person, and GHS (Globally Harmonized System of Classification, Labelling and Packaging of Chemicals) pictogram, for which we have labelled 267 images as our project-specific dataset. The original images are selected from footage recorded during project workshops as well as drone videos of firefighting missions from the internet. Figure 5 shows example images and labels of the dataset. Because our dataset is really small, we used transfer learning: the network was trained with COCO dataset first, and then fine-tuned with a mix of VOC dataset and our project-specific dataset.

**Figure 6:**
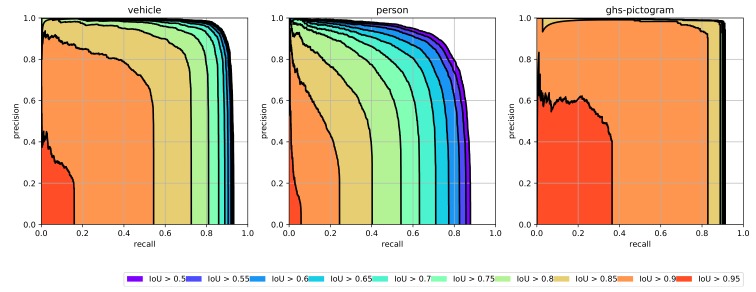
The precision–recall curve of object detection for individual classes.

**Figure 7:**
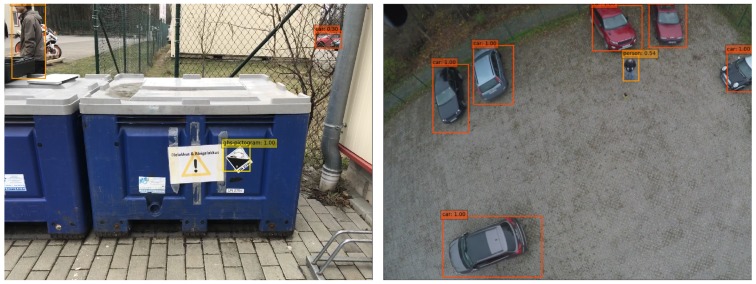
Object detection example: GHS (Globally Harmonized System of Classification, Labelling and Packaging of Chemicals) pictogram, person and vehicle. Number corresponds to confidence in [0,1].

According to our experiments on different backbones earlier, the sweet model with resnet50 as backbone was trained for our speed/accuracy requirements. Overall, the mAP of our solution is 0.875 for intersection over the union between prediction and ground truth bigger than 0.5. The Precision–Recall for individual classes is shown in Figure 6 with mAP for vehicle: 0.905, person: 0.811, and GHS pictogram: 0.909. Example images with detection results are presented in Figure 7.

### 4.2. Localisation and Navigation in the Transition of Indoor and Outdoor Environments

Today, the application of drones in firefighting scenarios is focusing on open areas that allow for GNSS-based control [1,2]. The problem is that, especially in large industrial sites, it would also be beneficial to inspect and explore shop floors, but indoor drones cannot rely on the GNSS signal. In EffFeu, we strive for an integrated approach that combines GNSS-based control with SLAM (Simultaneous Localisation and Mapping), prior known maps and other odometry information. Our approach is based on the experience we made in [31]. A challenge in our former approaches was the determination of the indoor world scale while using the monocular ORB-SLAM [32], for this reason we switched to the further continued version ORB-SLAM2 [33] that also supports RGB-D and stereo cameras to enable a direct determination of scale. For our scenario, we further extended ORB-SLAM2 (https://gitlab.tubit.tu-berlin.de/breakdowncookie/ORB_SLAM2). In particular, we integrated it into ROS, added additional interfaces, and developed a mapping component that generates an octree representation for navigation out of the sparse internal feature map of the algorithm. As an input sensor for SLAM, the RGB-D camera Intel^®^RealSense™ D435 is selected due to its low weight, small size, and outdoor functionality. In order to enable the application of the RGB-D sensor with the limited available computer power, we implemented an improved depth registration module. This performance optimised depth registration module enables us to use the RGB-D camera together with other modules in our system in contrast to the original version.

**Figure 8:**
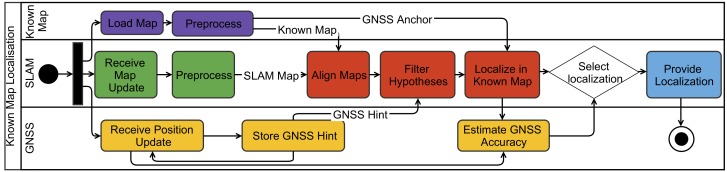
Localisation process combining information from prior known maps, SLAM and GNSS.

Moreover, we use a priori available ground plans with GNSS anchor to infer the absolute robot position through an alignment with SLAM generated maps. This is possible in our scenario because industrial firefighters are operating in known environments. Furthermore, our system allows for improving the accuracy and robustness through filtering with partially available GNSS signals, which is especially applicable during the transition between outdoor and indoor environment. In particular, the available 2D ground plans are preprocessed to get a similar visual representation as the SLAM maps that are projected to 2D before we align both maps with a map alignment algorithm. In case of partially available GNSS information, we use this to filter out implausible map alignments. Finally, the best-found alignment hypothesis and the GNSS anchor from the known map is used to estimate the global position of the drone. This decision is based on the quality of the current map alignment, the GNSS signal quality and the coverage of the SLAM map in relation to the known map. All the process steps are separated into individual software components that can be easily exchanged and configured. In consequence, this modular architecture allows for conveniently selecting particular algorithms for filtering and alignment or replacing and extending them in future. The entire localisation process is visualised in Figure 8.

Even though ORB-SLAM2 shows superior performance compared to other available SLAM approaches, it is possible to lose track. In order to recover from such situations and to get a faster but less accurate short term estimation, we use an additional high-speed camera, distance sensor, and visual odometry. In [31], we used the PX4Flow, but it turned out that we had to make various workarounds to get reasonable performance. For this reason, we implemented a comparable ROS module that runs directly on our companion computer based on [34].

#### 4.2.1. Experiments

In order to evaluate our localisation process in several complex settings with available known maps, GNSS signals and ground truth information we used the MORSE simulator [35] for which we created four environment models, two indoor and two outdoor scenes. Using a simulated environment has the advantage of providing a valid ground truth that would not be available in the necessary scale outdoors since we would need an external tracking system. In particular, we investigated which of the following approaches for the map alignment between the SLAM-generated map and the a priori available environment map provides the best performance in our use case. Furthermore, we tested if the implemented additional GNSS-filtering allows for improving the localisation result.

The first considered approach is called *mapmerge* [36] and uses Hough lines and the concept of Hough spectra to align two binary occupancy maps. The ROS package *mapstitch* (http://wiki.ros.org/mapstitch) extracts ORB features from the maps and calculates the alignment transformation via feature matching. Two further algorithms are from the ROS package *cs_merge* (http://wiki.ros.org/cs_merge) and are both variations of the Iterative Closest Point (ICP) algorithm. The first algorithm, *ICP gradient*, is based on [37] and *ICP SVD* is based on [38].

**Figure 9:**
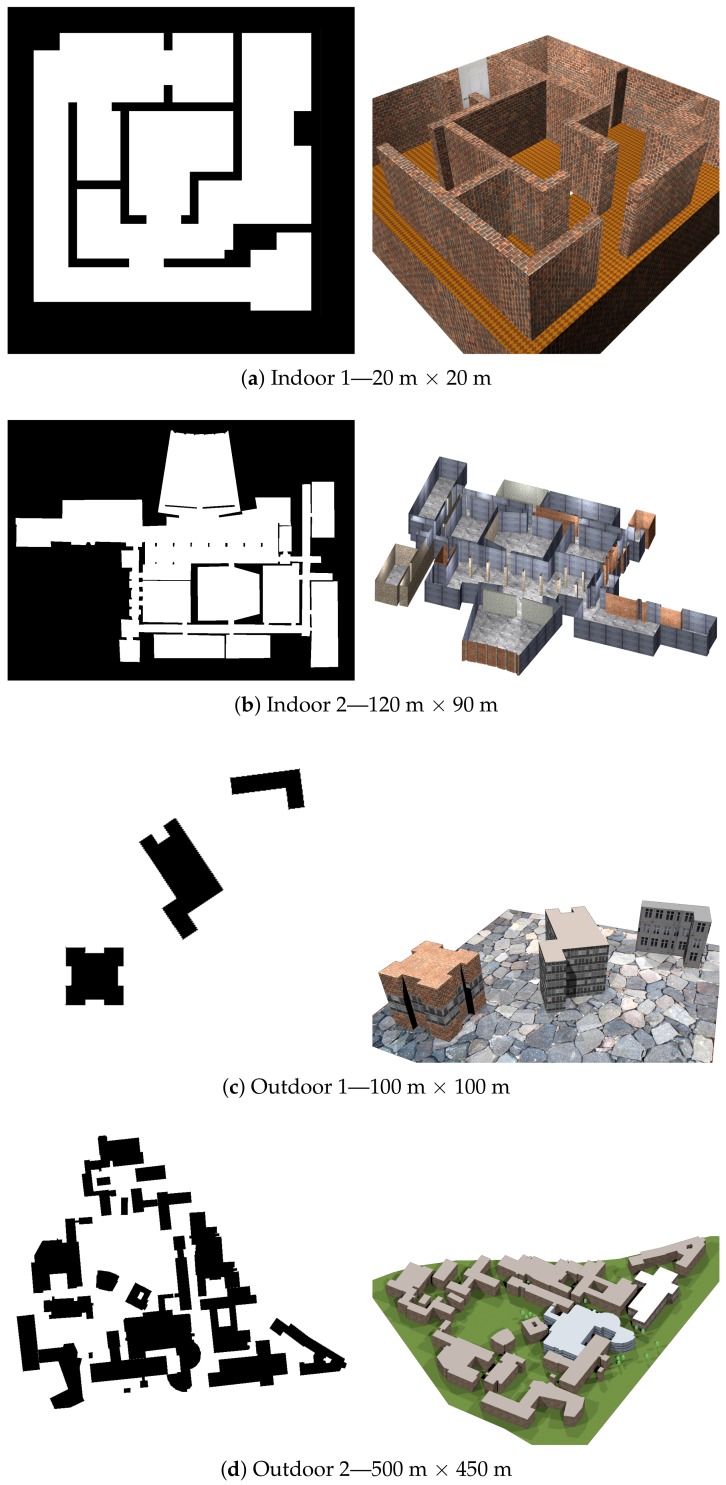
Known maps (**left**) and renderings (**right**) of simulation environments with their rough sizes. These occupancy maps show occupied (black) and free (white) regions.

The known maps we used for the experiments are shown in Figure 9 together with renderings of the corresponding simulation models.

The models are of different complexity and size, so that we could get an impression of how well our approach works in different environments. During the test flights with a UAV model, our estimated robot pose was logged as well as the ground truth data provided by the simulator. The trajectory of the drone is scripted and independent of the evaluated localisation.

#### 4.2.2. Discussion

**Figure 10:**
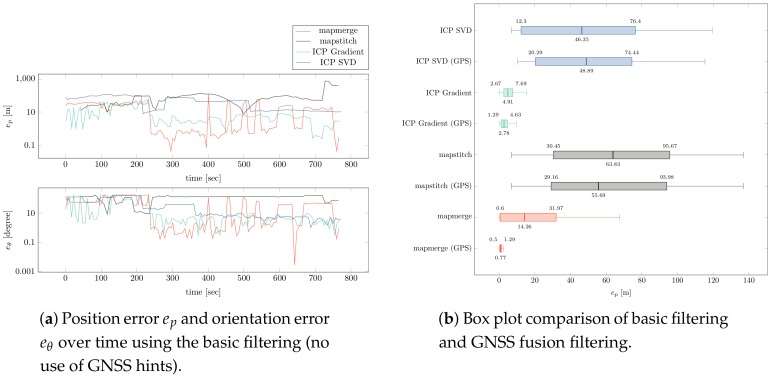
Position and orientation error plots for a simulated flight in the 100 m × 100 m sized *outdoor 1* environment.

The error, which is the deviation of the computed pose from the real pose, is shown in Figure 10. We split the pose error into position error $e_{p}$ and orientation error $e_{\theta }$. The plot over time shows that the error is strongly fluctuating. This is due to incorrect hypotheses being selected to compute the robot pose. Especially if no GNSS hints are available and only basic filtering is applied, the deviation is very high as visualised in the box plot. Additionally, not all alignment algorithms perform equally well. The *mapstitch* and *ICP SVD* algorithms did not find any approximately correct map alignments; therefore, even with the GNSS fusion filtering, the variance is very high. In the other cases, GNSS fusion was able to significantly lower the median error and reduce variance. A median $e_{p}$ of 0.77 meters as with *mapmerge* is a suitable accuracy for localization in larger outdoor environments. In small indoor environments (*indoor 1* in Figure 9), we even achieved a smaller median error $e_{p}$ of 0.3 meters and $e_{\theta }$ of 0.7 degrees.

Over the series of simulated test flights in different environments and with varied settings *mapmerge* turned out to be the most robust and reliable algorithm.

However, the localization accuracy of our approach highly depends on size and geometric complexity of the known map. Simple geometric shapes proved to be easier to match than cluttered maps. The resolution of maps also plays an important role as a low resolution obviously makes exact matching difficult.

For several reasons, the error goes up with the size of the mapped environment. One reason is that a small error in rotation of the alignment leads to bigger positional errors the further away the robot’s current position is from the rotation pivot. A second reason is SLAM drifts. In big SLAM maps, we are often facing a significant error due to drift over time. This leads to a SLAM map that is slightly distorted compared to the known map. Such a map might only locally match the known map, hence it is difficult to find a globally valid alignment.

### 4.3. Mission-Guided Control

Today’s control of drones is very much focused on the spatial control of the systems. Drones are either controlled manually or follow preprogrammed GNSS trajectories in an automated fashion. However, most professional users are not interested in the actual aerial system and which trajectories it has to fly in order to perform its task like collecting the required information. An alternative is a mission-guided control that defines the scope of operation for the drone with goal specifications. In order to apply goal specifications in an autonomous mission execution, we utilise decision-making (action selection) and planning.

#### 4.3.1. Decision-Making and Planning

In EffFeu, we use the task-level decision-making and planning framework *ROS Hybrid Behaviour Planner (RHBP)* [39] to implement the execution and decision-making in the EffFeu project. The RHBP framework builds on top of the ROS framework. The RHBP framework combines the advantages of reactive opportunistic decision-making and goal-oriented proactive planning in a hybrid architecture. The decision-making layer is based on the idea of behaviour networks that allow for dynamic state transitions and the definition of goals. The deliberative layer makes use of state-of-the-art planners through its Planning Domain Description Language (PDDL) interface. In particular, we integrated a further improved version of the planner Metric-FF [40], a version of FF extended by numerical fluents, support for optimization criteria and conditional effects. It meets all requirements and, due to its heuristic nature, favours fast results over optimality.

In RHBP, a problem is modelled with behaviours, preconditions, effects and goals, whereby conditions are expressed as a combination of virtual sensors and activation functions. Activation functions allow for a heuristical evaluation of the influence of particular information, which is gathered by a sensor, on the decision-making. The sensors are providing an abstract interface to arbitrary information sources such as ROS topics, the ROS parameter server, the RHBP knowledge base (a tuple space) or any other self-implemented information source. A behaviour represents any task or action a robot can execute which has an influence on the environment that is perceived by the sensors. Which abstraction level is chosen here is up to the user and depends as well on the application scenario. It is also possible to model higher-level behaviours that again consists of an own decision-making and planning model.

The actual operational drone behaviour is modelled and implemented on the foundation of RHBP base classes for goals, behaviours, sensors, activators, and conditions. These components allow for modelling a dependency network of conditions and effects between goals and behaviours, which results in a behaviour network. The activators are applied to interpret discretised sensor values for decision-making. The symbolic planning is automatically executed by the manager component after it has compiled a PDDL domain and problem description from the current behaviour network representation.

In RHBP, the planner is used to guide the behaviour network towards a goal supporting direction instead of forcing the execution of an exact plan; this fosters opportunistic behaviour of the robot. Moreover, this results in a very adaptive and reactive behaviour that is frequently updated by the current perception.

In detail, the actual behaviours are selected based on their current activation. The activation value is calculated from seven activation/inhibition sources: activation from the precondition satisfaction, activation from succeeding behaviours, activation from preceding behaviour, activation from goals, inhibition from conflicting goals, inhibition from conflicting behaviours, and activation from the planner. Each activation value from the sources describes how much a behaviour is supporting or disapproving a particular behaviour or goal relationship. The combination of the activation sources is a weighted sum that allows adjusting the influence of the specific activation sources in order to direct the decision-making into a certain direction. The seven activation sources result in six weights [0,1] because we use the same weight for both inhibition sources. The influence of the activation sources respectively weights is described in the following. The meaning of larger and smaller weight values has to be considered always in respect to other weights.

The activation from preconditions only depends on the environment. It is completely independent from other behaviours and their activation. The activation from preconditions describes the level of fulfilment of the preconditions of a behaviour. The corresponding *situation weight* allows for striving for fast reactions to environmental changes, more opportunistic and egocentric decisions with larger values in comparison to other behaviour-dependent activation weights. Too large values can be problematic if a certain order of goals needs to be guaranteed.

The *predecessor weight* can be used to adjust the influence of executable predecessors on their successors. It supports to spread opportunities from the bottom up (from the perception in direction of the goals) and activates behaviours whose preconditions are most likely fulfilled in the future.

The *successor weights* accounts for the positive influence of a behaviour to its successors independent of a goal. The successor weight influences the thrustfulness of a network path. Due to only positive effects being considered, too large values might lead to the execution of behaviours that are perhaps regretted in the future.

The inhibition sources for behaviours and goals as well as the corresponding *conflictor weight* are used to prevent or reduce undesirable situations. A large conflictor weight results in more cautious and slow decisions, while too large values might result in decisions leading to a dead end of unpopular behaviours and prevents to reach certain goals.

The *goal weight* emphasises a goal-driven character of decision-making with larger values, while it encounters the risk of being too opportunistic so that it favours goal-fulfilling but otherwise mission-breaking behaviours.

It is important to remark that goal weight and successor weight both support activation flow towards the goals, although they are very different. The goal weight acts only on behaviours directly contributing to (or conflicting with) goals, while the successor weight also affects intermediate behaviours.

As already stated above the symbolic planner and corresponding *planner weight* are used to guide the behaviour network component with the determined sequential plan of the particular PDDL planner. A larger value results in a more dominant influence, while a smaller value will give only little guidance. Applying the planner allows supporting a certain order of behaviour but relies on the existence of plans, which is not always possible or feasible in time in the considered dynamic environments.

#### 4.3.2. End-User and UI Integration

**Figure 11:**
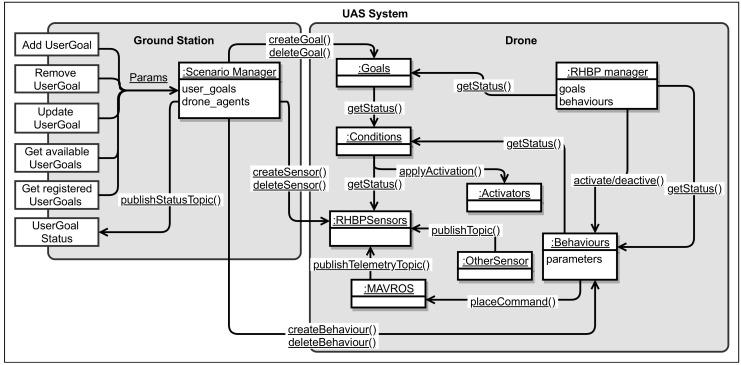
Planner component relationships and external user goal Application Programming Interface (API) in Unified Modeling Language (UML) communication diagram style without particular communication sequence. Arrows show messages and directions. Plural names of object instances indicate that multiple objects of this type are possible.

In EffFeu, the user interfaces are connected to RHBP through an external goal API that lists all available goals and possible parametrisations. Subsequently, the user interface allows for activating and configuring goals on demand through the external goal API. The high-level goals that are enabled by the user are mapped to corresponding RHBP goals that are automatically instantiated or configured by the interface component. Here, it is also possible that a single goal from the user perspective, like following a person, is internally decomposed to several RHBP components. We differentiate such user goals from the internal RHBP goal components. A user goal might be, for instance, a combination of internal safety goals considering the battery consumption and the actual goal of holding a certain distance to the tracked object. Moreover, in some cases, user goals are a composition of RHBP goals, behaviours, and sensors’ instances to be able to parametrise and configure the behaviours. This is necessary because RHBP does not support passing of parameters on the logical planning level, which are required for a behaviour to be executable. In consequence, running behaviour instances that require additional information like the flight destination need to be dynamically instantiated or adjusted during runtime independent from RHBP’s plan execution. In the EffFeu scenario, we dynamically create instances of the behaviours with the goal specific parameters because altering existing instances is problematic as the goals are allowed to be instantiated multiple times. For other more abstract behaviours such as take-off, landing, and collision avoidance, the corresponding user goal does only contain goals that consist of conditions formed with RHBP sensors and activators. The detailed relationships and communications of the so-called *Planner* component introduced in Section 3 is visualised in Figure 11. Furthermore, the diagram highlights that the management of the user goals is handled in a scenario, respectively an application specific manager component, which is responsible for creating or deleting the RHBP components that are used to model the achievement of a certain goal.

#### 4.3.3. Milestone Scenario

**Figure 12:**
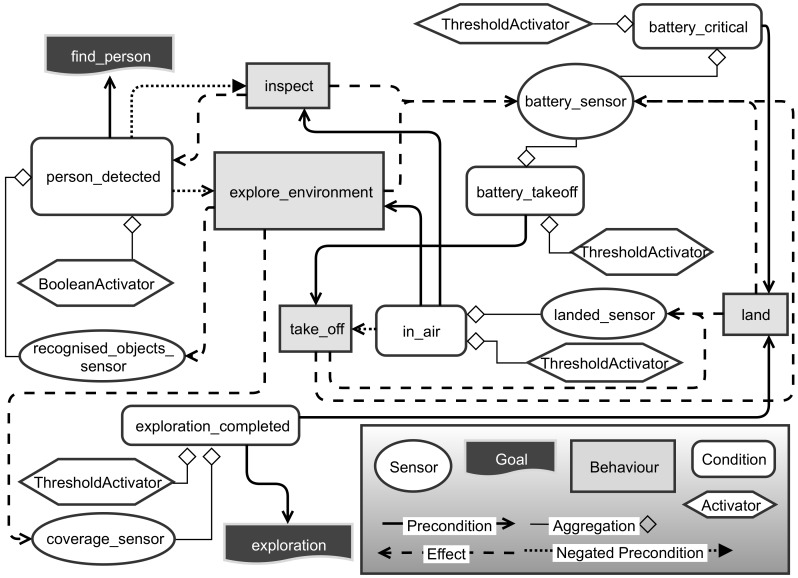
Behaviour network model of the milestone demonstrator scenario.

A first simple example of the application of RHBP is illustrated in Figure 12. The shown behaviour model was used as the mid-project milestone demonstrator as the first proof of concept. In the scenario, the mission goal is to explore a selected environment and once a person is found to circumfly (inspect) the found person to gather more information about the situation. To model this scenario, we use two goals, one for initiating the exploration behaviour and one for detecting a person. The *recognised_objects_sensor* connects to the results of our object detection, whereas the *coverage_sensor* describes the exploration completion of the selected area percentagewise. Additionally, the implemented behaviour model considers the current battery level of the drone to safely land in case of an empty battery or prevent mission launches without sufficient battery.

Undoubtedly, the given scenario is comparably simple and would not require a complex planning and decision-making system. Nevertheless, the implemented model performed well in a qualitative evaluation during the milestone demonstration, where the drone showed the intended behaviour after the corresponding user goal was created. Creating the goal was both demonstrated through the *Mission-Control-UI* as well as the *Safety and Security Management System* of our partners. The intention of this example is to illustrate the application and integration options with a simple scenario. A further developed system being equipped with more different behaviours and other goal sets is described and evaluated in the following section.

#### 4.3.4. Evaluation: Dynamic Scenario

The milestone scenario explained in the last section is statically initialised and executed without showing the benefits of such an approach in a dynamic environment. In particular, different behaviours that can be used to achieve the same result but different in their particular characteristics, such as specific preconditions and effects, as well as changing the mission goal during runtime, make the application of a planning component meaningful because it allows for efficiently choosing the most suitable behaviour depending on the current situation. In the following, we will explore such more complex and dynamic application scenarios that have been realised after the project milestone.

The complex scenario comprises some changes with respect to the milestone scenario. First, the main task of the scenario is modified in the way that now finding a person and inspecting the environment as well as exploring the given area for the purpose of creating a map have the same priority. This is in contrast to the milestone scenario wherein the exploration is only necessary to find the person.

Secondly, additional possible behaviours have been integrated, namely *arm* for arming the drone automatically, *idle* to model an option of doing nothing on the ground, *hover* to wait in the air, and two alternative exploration behaviours. The exploration behaviours differ in the exploration speed and the quality of exploration. The slow exploration is traversing the area of interest in parallel lines with a smaller distance and higher overlap in comparison to the fast exploration. This difference results in a longer flight trajectory for the slow exploration but a higher probability of finding objects with object recognition. The *remember_position* is an example of a behaviour that is only affecting internal system states by storing the position of the person after it has been found.

**Figure 13:**
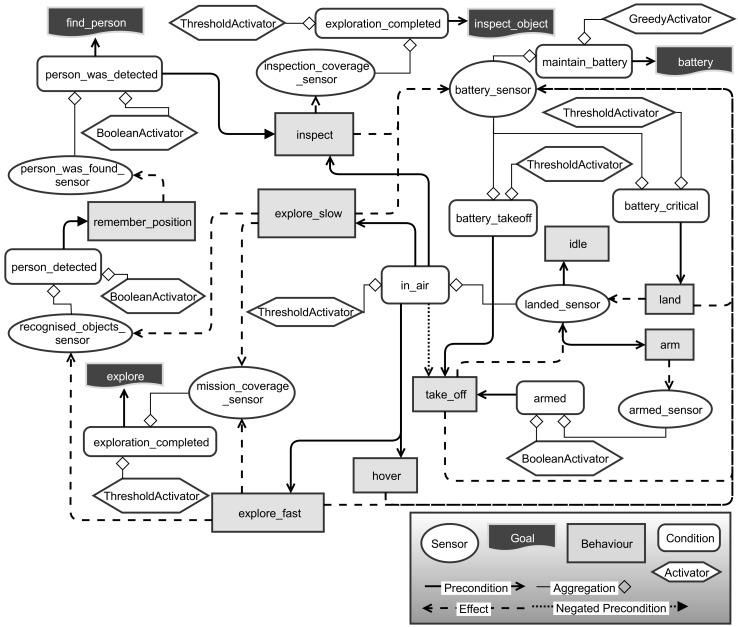
More complex behaviour network model including more and alternative behaviours for similar effects and additional goals.

The new behaviour network model is illustrated in Figure 13. The diagram shows also that we added additional goals to improve the overall mission execution. The *exploration goal* is fulfilled by completing the exploration of the area of interest. This can be potentially achieved by running both exploration behaviours; however, they have been modelled with different effect intensities to represent the above-described differences—in detail, *exploration_slow* has a greater influence on the *recognised_objects_sensor* and a smaller influence on the progress of the *mission_coverage_sensor*. The *inspect_object goal* is achievable by inspecting the area around the found object.

The additional *battery goal* is a maintenance goal that tries to minimise the usage of the battery. Here, the so-called *GreedyActivator* of RHBP is used to model a condition that is never satisfied and always aiming for a maximisation of the particular sensor.

In order to evaluate the complex behaviour model, we are using a simulation environment that we implemented with the generic Morse simulator [35]. The simulation environment has been extended to mimic the same ROS API as we are using on the real drone for external control. The original API in use is a combination of the MAVLink protocol of the PX4 firmware and the MAVROS package for the automated creation of ROS bindings. This approach allows us to easily test our implementations before they can be seamlessly transferred to the actual hardware. This works especially well for the mission-guided control because here we are mostly interested in the high-level decision made and not in the detailed motion of the drone, which is not simulated precisely. Using a simulator for evaluating the higher-level behaviour has the advantage of always having the same conditions.

A difference to former applications of the RHBP framework is that in the EffFeu project goals are dynamically created, enabled and disabled during runtime through the above-described external user interface. Aside from simple test scenarios in [39,41], RHBP has so far been used only with a single drone in a static mission of the SpaceBot Cup [31], as well as with static mission goals in simulated multi-robot scenarios in the Multi-Agent Programming Contest [42,43].

In the following experiments, we wanted to see if our framework is able to properly handle the adaptation to dynamically enabled/disabled goals. Moreover, we investigated to what extent the influence of the symbolic planner in our hybrid approach is supporting the overall mission accomplishment and adaptation in such scenarios. Particularly, we analysed the influence on the efficiency, how fast the mission is accomplished, as well as the adaptation capabilities, and how fast is the system reacting to environmental and mission changes.

In order to examine the influence of dynamically created, enabled and disabled goals, we have scripted a scenario within the simulation environment for the above described behaviour model. The scenario starts with the goals *find_person*, *explore*, and *battery* enabled. After 18 decision-making steps, the *find_person* and *explore* goal are disabled and enabled again after step 24. An additional *inspect_object* goal is then created after step 30. In this scenario, all goals except for the *battery* goal are achievement goals that are removed after they are completed. Moreover, environmental changes, respectively, perception changes are simulated through the suddenly detected target object.

For the investigation of the influence of the symbolic planner in such dynamic scenarios, we repeated the experiment with different weights for the influence of the symbolic planner, which have to be in a range of 0 to 1. Furthermore, we limited RHBP to enable only one behaviour at a time because, in the given setup, behaviours would heavily conflict with each other by sending different low-level control commands at the same time. Other activation weights: activation by situation, predecessors, successors, and conflictors (see [39] for details) that are used in the RHBP decision-making are set to a medium influence of 0.5 except for the activation by goals that we empirically configured to 0.7 to foster goal pursuance in all experiments. We have fixed the weights except the planner weight to empirically determined values because we are especially interested in the influence of the planner as a major component of our hybrid approach. All other weights are only relevant for the decision-making of the behaviour network layer itself and the fine-tuning of its decision-making, which influences the stability but not the overall adaptation means.

**Figure 14:**
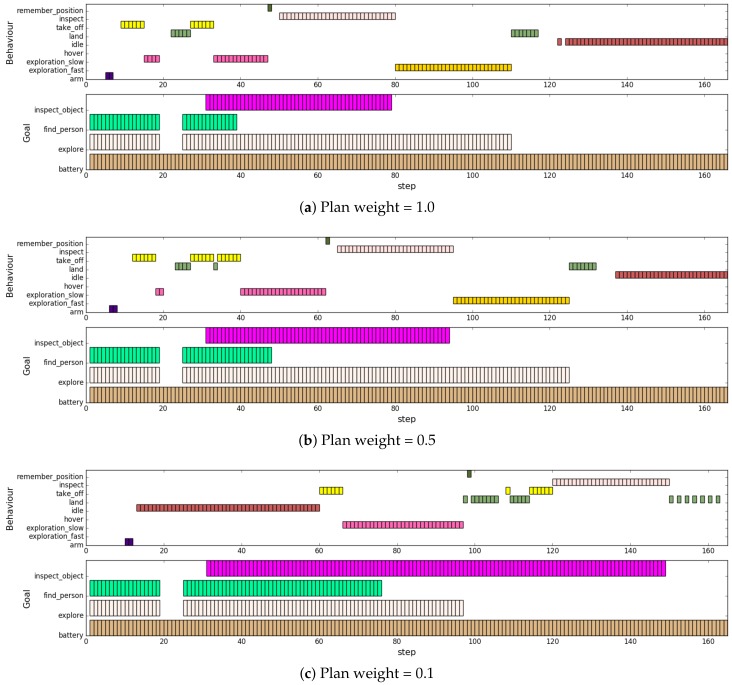
Selected behaviours and enabled goals over time ($\frac{\mathrm{step}}{s}$). Comparison between different characteristic symbolic planning influences specified by weights.

The diagrams in Figure 14 visualise a selection of the most characteristic experiment runs with logged decisions and corresponding goals. The drawn bars describe which behaviour is selected for execution as well as which goals are enabled in a particular decision-making step. We decided to show the run with very little symbolic planning influence (weight = 0.1) instead of the run without influence. The reason is that both result sequences are very similar but with weight = 0.0 the complete execution takes just longer (230 decision steps), which makes it more difficult to compare the diagram with the other results. Decision steps without a decision taken are possible if the current sensor’s respective drone state together with precondition setup do not allow for any behaviour to be activated. This is sometimes occurring due to some not in the behaviour model specified drone states when the drone is in the transition between being in-air and landed.

In general, the results show that, in all configurations, the given mission is completed after some time. Furthermore, RHBP is able to handle dynamically created or enabled and disabled goals during runtime (see (a) and (b)). The goal pursuance of the system without (much) influence from the planner (c) is not sufficient, the system is having problems to handle the conflicting goals of maintaining the battery and completing all other tasks, which require battery capacity. This becomes visible already in (b) where the system suddenly decides for landing one time after step 30. In (c), this is more obvious because, without the additional *inspect_goal*, the overall activation of the battery consuming goals is not high enough to start the overall mission. Alternative exploration behaviours are correctly selected in (a) and (b) depending on the currently available boundary conditions. First, the *exploration_slow* is selected until the person is found. After the person is found, the *exploration_fast* is favoured to fasten the exploration. Adaptation capabilities in the sufficiently completed missions of (a) and (b) are indicated by the number of required steps after the goals are activated, deactivated, completed, and time to consider the detected person. Both configurations (a) and (b) show a very similar performance. The adaptation time to the found person is two steps, to reactivated goals (find_person, explore) two steps, and to completed goals (inspect_object, explore) one step for both (a) and (b). Only the response to the temporary deactivation of the goals (find_person, explore) at the beginning of the mission is slightly different. Here, configuration (a) is adapting after three steps, while (b) requires four steps.

Greater influence from the planner fosters the efficiency, the entire mission is completed in a shorter time, (a) is faster than (b), and (b) is faster than (c). The adaptation capabilities, once the goals are sufficiently pursued, are not much affected by the influence of the symbolic planner. Reducing the influence of the planner makes decision-making less stable, and see unnecessary take-offs and landings in (c). Even though a more stable decision-making could also be achieved by tuning the model and weights more carefully, the result would depend on the particular scenario implementation and would have to be revised every time when the implementation is changed.

Results underpin that the hybrid RHBP approach, which applies long-term planning to direct the short-term decision making, is reasonable to foster the goal pursuance of the system as well as the efficiency. Furthermore, influence of the symbolic planner is especially useful in situations of conflicting and dynamically changed goals, whereas influence on adaptation time is negligible.

Furthermore, as our results indicate the usefulness of long-term planning within a hybrid approach, where the planner is guiding the short-term decision-making, it would also be interesting to conduct research about an alternative inverted approach, where long-term planning is guided by the weights of short-term decision-making as some kind of utility function.

## 5. Conclusions

The EffFeu project developed the foundation to make the integration of UAVs into the safety and security management system easy and easy to use. The efficient use of drones in the daily routine of industrial firefighters and in case of emergency is still challenging. With our system under development, firefighters can already benefit from a mission-guided control and enhanced autonomy of the system in known indoor and outdoor environments that reduces their cognitive load. They can already assign mission goals with priorities to drones, which are then reassembled and planned as sequences of actions by the drones. Further autonomy in object recognition and situation assessment has been added to the drone to automate the analysis of recorded video footage. All together, this already enables a transition from motion and trajectory-based drone control towards a goal and mission-oriented control and application in the operation of autonomous drones.

The next steps will include work on object tracking (preserve the object identity over consecutive images, object lost regain) and situation assessment (analyse the temporal, and spatial object environment and context, correlations between objects). Additionally, it is planned to apply user feedback about false positives to improve the object recognition during runtime. Moreover, we will extend the decision-making and planning model over multiple drones and combine it with self-organisation concepts for decentralised coordination based on our work presented in [41]. Additionally, the realised localisation approach will be further analysed in qualitative tests in real mission environments.

The work will flow into a complex demonstrator, which shows the full potential of our approach and which will undergo extensive tests by industrial firefighters.

## Figures and Tables

**Table 1:** Benchmark of different backbone network trained on COCO dataset.

Backbone Net	No. of Parameters	Inference Time	mAP@0.5	mAP@0.5:0.95
mobilenet v2	12 M	110 ms	0.4884	0.2986
vgg16	47 M	200 ms	0.4911	0.3094
resnet50	47 M	160 ms	0.5211	0.3346
resnet101	72 M	180 ms	0.5343	0.3476
resnext101_32x4d	72 M	210 ms	0.5348	0.3501
se_resnext50_32x4d	56 M	200 ms	0.5257	0.3334
se_resnext101_32x4d	76 M	240 ms	0.5405	0.3553
senet154	142 M	370 ms	0.5461	0.3619
